# Bis[μ-*N*-(pyridin-2-yl)methane­sulfon­amido-κ^2^ *N*:*N*′]silver(I)

**DOI:** 10.1107/S1600536813031814

**Published:** 2013-11-27

**Authors:** Hui-Ling Hu, Chun-Wei Yeh

**Affiliations:** a Department of Hospitality Management, Taoyuan Innovation Institute of Technology, Jhongli 32091, Taiwan; b Department of Chemistry, Chung-Yuan Christian University, Jhongli 32023, Taiwan

## Abstract

In the title compound, [Ag_2_(C_6_H_7_N_2_O_2_S)_2_], the Ag^I^ atom is coordinated by two N atoms from two *N*-(pyridin-2-yl)methane­sulfonamidate anions in a slightly bent linear geometry [N—Ag—N = 166.03 (7)°]. The Ag^I^ atoms are bridged by the *N*-(pyridin-2-yl)methane­sulfonamidate anions, forming a centrosymmetric dinuclear mol­ecule, in which the Ag⋯Ag distance is 2.7072 (4) Å.

## Related literature

For related di(pyrid­yl/pyrimid­yl)amide structures, see: Hu *et al.* (2004 ▶); Hsu *et al.* (2008 ▶); Yeh *et al.* (2008 ▶); Tsai *et al.* (2010 ▶). For related methyl-4-(pyridin-pyrimidin-2-ylcarbamo­yl)benz­o­ate structures, see: Wu *et al.* (2011 ▶); Hsiao *et al.* (2012 ▶). For related phosphinic amide structures, see: Yeh & Chen (2011 ▶); Yeh *et al.* (2012 ▶).
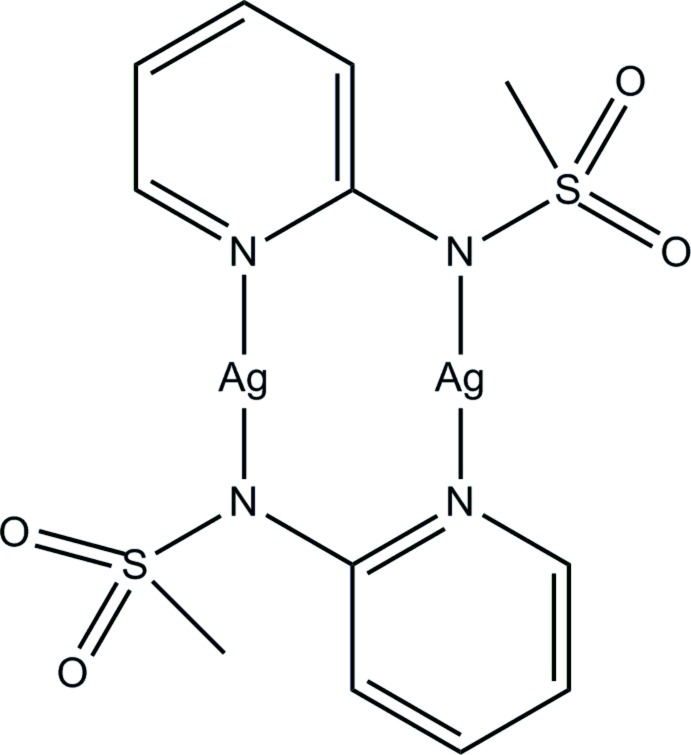


## Experimental

### 

#### Crystal data

[Ag_2_(C_6_H_7_N_2_O_2_S)_2_]
*M* *_r_* = 558.13
Monoclinic, 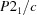

*a* = 6.4406 (2) Å
*b* = 15.4580 (5) Å
*c* = 8.0789 (2) Å
β = 97.143 (2)°
*V* = 798.08 (4) Å^3^
*Z* = 2
Mo *K*α radiation
μ = 2.74 mm^−1^
*T* = 296 K
0.20 × 0.10 × 0.10 mm

#### Data collection

Bruker APEXII CCD area-detector diffractometer
Absorption correction: multi-scan (*SADABS*; Bruker, 2000 ▶) *T*_min_ = 0.636, *T*_max_ = 0.747
12917 measured reflections
3569 independent reflections
2732 reflections with *I* > 2σ(*I*)
*R*_int_ = 0.048

#### Refinement

*R*[*F* ^2^ > 2σ(*F* ^2^)] = 0.036
*wR*(*F* ^2^) = 0.092
*S* = 1.05
3569 reflections
110 parameters
H-atom parameters constrained
Δρ_max_ = 1.32 e Å^−3^
Δρ_min_ = −1.22 e Å^−3^

### 

Data collection: *APEX2* (Bruker, 2010 ▶); cell refinement: *SAINT* (Bruker, 2010 ▶); data reduction: *SAINT*; program(s) used to solve structure: *SHELXS97* (Sheldrick, 2008 ▶); program(s) used to refine structure: *SHELXL97* (Sheldrick, 2008 ▶); molecular graphics: *DIAMOND* (Brandenburg, 1999 ▶); software used to prepare material for publication: *SHELXL97*.

## Supplementary Material

Crystal structure: contains datablock(s) I, New_Global_Publ_Block. DOI: 10.1107/S1600536813031814/xu5753sup1.cif

Structure factors: contains datablock(s) I. DOI: 10.1107/S1600536813031814/xu5753Isup2.hkl

Additional supplementary materials:  crystallographic information; 3D view; checkCIF report

## Figures and Tables

**Table 1:** Selected bond lengths (Å)

Ag—N1	2.1373 (19)
Ag—N2^i^	2.1654 (19)

Symmetry code: (i) 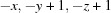.

**Fig. 1.**
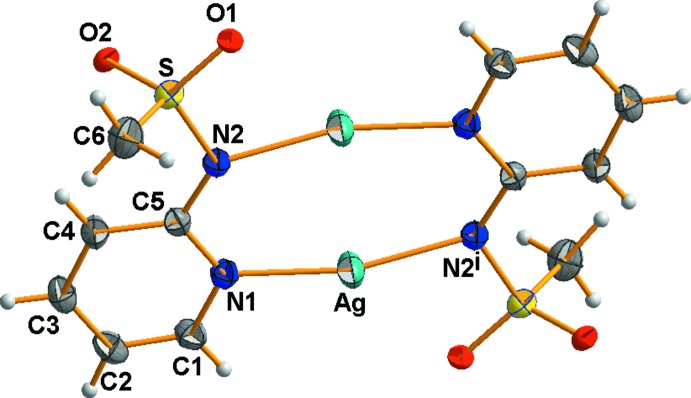
Crystal structure of the title compound with labeling and displacement ellipsoids drawn at 30% probability level. [Symmetry codes: (i) -x,-y + 1,-z + 1.]

## supplementary crystallographic information

### 0.1. Synthesis and crystallization

An aqueous solution (5.0 ml) of AgNO_3_ (1.0 mmol) was layered carefully over a methano­lic solution (5.0 ml) of N-(pyridin-2-yl)methansulfonamide (1.0 mmol) in a tube and kept it in the dark. Colourless crystals were obtained after several weeks. These were washed with methanol and collected in 78.6% yield.

### 0.2. Refinement

H atoms were placed in idealized positions and constrained to ride on their parent atoms with C—H = 0.93 or 0.96 Å, U_iso_(H) = 1.5U_eq_(C) for methyl H atoms and 1.2U_eq_(C) for the others.

### 1. Results and discussion

A series of complexes with the symmetric di(pyridyl/pyrimidyl)amide ligands (Hu *et al.*, 2004; Hsu *et al.*, 2008; Yeh *et al.*, 2008; Tsai *et al.*, 2010) and the asymmetric methyl-4-(pyridin-/pyrimidin-2-ylcarbamoyl)benzoate (Wu *et al.*, 2011; Hsiao *et al.*, 2012) or phosphinic amide (Yeh & Chen, 2011; Yeh *et al.*, 2012) ligands that exhibit inter­esting structural types have been synthesized and structurally characterized. These pyridyl/pyrimidyl amide ligands coordinate to the metal centers through their pyridyl/pyrimidyl nitro­gen atoms and/or amide oxygen atoms and inter­act with each other through hydrogen bonds involving the amide groups. These inter­actions are important for molecular recognition and constructing supra­molecular arrays. .

In the title compound, [Ag(C_6_H_7_N_2_SO_2_)]_2_, the Ag^+^ cations are coordinated with one pyridyl N and one amido N atoms from two*N*-(pyridin-2-yl)methane­sulfonamido (*L^-^*) anions forming a slightly bent geometry (Fig. 1). The Ag···Ag distance separated by the bridging *L^-^* group is 2.7072 (4) Å.

### Crystal data

**Table:**

[Ag_2_(C_6_H_7_N_2_O_2_S)_2_]	*F*(000) = 544
*M**_r_* = 558.13	*D*_x_ = 2.323 Mg m^−^^3^
Monoclinic, *P*2_1_/*c*	Mo *K*α radiation, λ = 0.71073 Å
Hall symbol: -P 2ybc	Cell parameters from 6474 reflections
*a* = 6.4406 (2) Å	θ = 2.6–35.3°
*b* = 15.4580 (5) Å	µ = 2.74 mm^−^^1^
*c* = 8.0789 (2) Å	*T* = 296 K
β = 97.143 (2)°	Column, colourless
*V* = 798.08 (4) Å^3^	0.20 × 0.10 × 0.10 mm
*Z* = 2	

### Data collection

**Table:**

Bruker APEXII CCD area-detector diffractometer	3569 independent reflections
Radiation source: fine-focus sealed tube	2732 reflections with *I* > 2σ(*I*)
Graphite monochromator	*R*_int_ = 0.048
phi and ω scans	θ_max_ = 35.4°, θ_min_ = 2.6°
Absorption correction: multi-scan (*SADABS*; Bruker, 2000)	*h* = −10→8
*T*_min_ = 0.636, *T*_max_ = 0.747	*k* = −25→22
12917 measured reflections	*l* = −13→11

### Refinement

**Table:**

Refinement on *F*^2^	Primary atom site location: structure-invariant direct methods
Least-squares matrix: full	Secondary atom site location: difference Fourier map
*R*[*F*^2^ > 2σ(*F*^2^)] = 0.036	Hydrogen site location: inferred from neighbouring sites
*wR*(*F*^2^) = 0.092	H-atom parameters constrained
*S* = 1.05	*w* = 1/[σ^2^(*F*_o_^2^) + (0.0368*P*)^2^ + 0.3136*P*] where *P* = (*F*_o_^2^ + 2*F*_c_^2^)/3
3569 reflections	(Δ/σ)_max_ = 0.001
110 parameters	Δρ_max_ = 1.32 e Å^−^^3^
0 restraints	Δρ_min_ = −1.22 e Å^−^^3^

### Special details

**Table:**

Geometry. All e.s.d.'s (except the e.s.d. in the dihedral angle between two l.s. planes) are estimated using the full covariance matrix. The cell e.s.d.'s are taken into account individually in the estimation of e.s.d.'s in distances, angles and torsion angles; correlations between e.s.d.'s in cell parameters are only used when they are defined by crystal symmetry. An approximate (isotropic) treatment of cell e.s.d.'s is used for estimating e.s.d.'s involving l.s. planes.
Refinement. Refinement of *F*^2^ against ALL reflections. The weighted *R*-factor *wR* and goodness of fit *S* are based on *F*^2^, conventional *R*-factors *R* are based on *F*, with *F* set to zero for negative *F*^2^. The threshold expression of *F*^2^ > σ(*F*^2^) is used only for calculating *R*-factors(gt) *etc*. and is not relevant to the choice of reflections for refinement. *R*-factors based on *F*^2^ are statistically about twice as large as those based on *F*, and *R*- factors based on ALL data will be even larger.

### Fractional atomic coordinates and isotropic or equivalent isotropic displacement parameters (Å^2^)

**Table:**

	*x*	*y*	*z*	*U*_iso_*/*U*_eq_	
Ag	0.13566 (3)	0.475803 (15)	0.63351 (2)	0.03999 (8)	
S	0.07511 (10)	0.64837 (4)	0.14689 (7)	0.02803 (12)	
C1	0.5244 (4)	0.58574 (16)	0.6741 (3)	0.0306 (4)	
H1A	0.5410	0.5553	0.7742	0.037*	
C2	0.6811 (4)	0.64120 (18)	0.6425 (3)	0.0354 (5)	
H2A	0.8011	0.6483	0.7181	0.043*	
C3	0.6530 (4)	0.68622 (16)	0.4931 (3)	0.0357 (5)	
H3A	0.7560	0.7241	0.4664	0.043*	
C4	0.4738 (4)	0.67511 (16)	0.3846 (3)	0.0318 (5)	
H4A	0.4547	0.7060	0.2851	0.038*	
C5	0.3186 (3)	0.61698 (14)	0.4237 (3)	0.0245 (4)	
C6	0.2438 (5)	0.6112 (2)	0.0057 (3)	0.0422 (6)	
H6A	0.2090	0.6393	−0.1001	0.063*	
H6B	0.2281	0.5498	−0.0083	0.063*	
H6C	0.3860	0.6243	0.0487	0.063*	
N1	0.3482 (3)	0.57273 (12)	0.5690 (2)	0.0255 (3)	
N2	0.1362 (3)	0.59867 (13)	0.3207 (2)	0.0274 (4)	
O1	−0.1324 (3)	0.61682 (13)	0.0849 (2)	0.0406 (4)	
O2	0.1003 (3)	0.74049 (12)	0.1604 (3)	0.0402 (4)	

### Atomic displacement parameters (Å^2^)

**Table:**

	*U*^11^	*U*^22^	*U*^33^	*U*^12^	*U*^13^	*U*^23^
Ag	0.03197 (12)	0.05057 (14)	0.03650 (11)	−0.01463 (8)	0.00059 (8)	0.01295 (8)
S	0.0278 (3)	0.0277 (3)	0.0283 (3)	0.0004 (2)	0.00232 (19)	0.00570 (19)
C1	0.0274 (11)	0.0352 (12)	0.0286 (10)	−0.0003 (9)	0.0011 (8)	−0.0004 (8)
C2	0.0271 (12)	0.0397 (13)	0.0381 (13)	−0.0043 (10)	−0.0016 (9)	−0.0069 (10)
C3	0.0319 (12)	0.0328 (11)	0.0426 (13)	−0.0105 (10)	0.0054 (10)	−0.0031 (10)
C4	0.0300 (12)	0.0317 (11)	0.0337 (11)	−0.0076 (9)	0.0040 (9)	0.0033 (9)
C5	0.0227 (10)	0.0246 (9)	0.0267 (9)	−0.0013 (7)	0.0047 (7)	−0.0011 (7)
C6	0.0492 (17)	0.0462 (15)	0.0330 (12)	−0.0013 (12)	0.0125 (11)	−0.0002 (10)
N1	0.0251 (9)	0.0272 (9)	0.0245 (8)	−0.0005 (7)	0.0041 (6)	0.0009 (6)
N2	0.0242 (9)	0.0306 (9)	0.0272 (8)	−0.0035 (7)	0.0017 (7)	0.0060 (7)
O1	0.0325 (10)	0.0472 (11)	0.0388 (10)	−0.0050 (8)	−0.0082 (8)	0.0109 (8)
O2	0.0416 (11)	0.0275 (8)	0.0506 (11)	0.0033 (7)	0.0028 (9)	0.0078 (7)

### Geometric parameters (Å, º)

**Table:**

Ag—N1	2.1373 (19)	C2—H2A	0.9300
Ag—N2^i^	2.1654 (19)	C3—C4	1.370 (4)
Ag—Ag^i^	2.7072 (4)	C3—H3A	0.9300
S—O2	1.4359 (19)	C4—C5	1.409 (3)
S—O1	1.452 (2)	C4—H4A	0.9300
S—N2	1.6061 (19)	C5—N1	1.351 (3)
S—C6	1.766 (3)	C5—N2	1.382 (3)
C1—N1	1.345 (3)	C6—H6A	0.9600
C1—C2	1.372 (4)	C6—H6B	0.9600
C1—H1A	0.9300	C6—H6C	0.9600
C2—C3	1.385 (4)	N2—Ag^i^	2.1654 (19)
			
N1—Ag—N2^i^	166.03 (7)	C3—C4—C5	120.1 (2)
N1—Ag—Ag^i^	88.95 (5)	C3—C4—H4A	120.0
N2^i^—Ag—Ag^i^	80.07 (5)	C5—C4—H4A	120.0
O2—S—O1	116.73 (12)	N1—C5—N2	115.93 (19)
O2—S—N2	113.24 (11)	N1—C5—C4	119.3 (2)
O1—S—N2	104.81 (11)	N2—C5—C4	124.7 (2)
O2—S—C6	107.40 (13)	S—C6—H6A	109.5
O1—S—C6	106.38 (14)	S—C6—H6B	109.5
N2—S—C6	107.79 (13)	H6A—C6—H6B	109.5
N1—C1—C2	123.9 (2)	S—C6—H6C	109.5
N1—C1—H1A	118.0	H6A—C6—H6C	109.5
C2—C1—H1A	118.0	H6B—C6—H6C	109.5
C1—C2—C3	117.2 (2)	C1—N1—C5	119.3 (2)
C1—C2—H2A	121.4	C1—N1—Ag	117.82 (15)
C3—C2—H2A	121.4	C5—N1—Ag	122.74 (15)
C4—C3—C2	120.2 (2)	C5—N2—S	121.75 (15)
C4—C3—H3A	119.9	C5—N2—Ag^i^	130.60 (14)
C2—C3—H3A	119.9	S—N2—Ag^i^	106.75 (10)

Symmetry code: (i) −*x*, −*y*+1, −*z*+1.
